# Dietary Sodium-Potassium Imbalance and Hypertension: Causal Pathways Involving Gut Microbiota Dysbiosis, Inflammation, and Metabolic Disorders

**DOI:** 10.31083/RCM44058

**Published:** 2025-12-18

**Authors:** Chuan Lu, Jiaxi Sun, Yue Zhang, Xin Zhao

**Affiliations:** ^1^Department of Cardiology, The Second Hospital of Dalian Medical University, 116021 Dalian, Liaoning, China; ^2^Department of Gastroenterology, The Second Hospital of Dalian Medical University, 116021 Dalian, Liaoning, China

**Keywords:** hypertension, urinary sodium-to-potassium ratio, Mendelian randomization analysis, inflammation, gut microbiota, metabolic disorder

## Abstract

**Background:**

The urinary sodium-to-potassium (UNa/UK) ratio reflects the dietary sodium and potassium balance and may serve as a biomarker for hypertension (HTN). An imbalance in the dietary sodium–potassium ratio may contribute to systemic inflammation, alterations in gut microbiota (GM), and related metabolic disorders. This study aimed to investigate the relationship between the UNa/UK ratio, HTN, inflammation, GM, and metabolic abnormalities using cross-sectional and Mendelian randomization (MR) analyses.

**Methods:**

We included 1210 hospitalized patients (median age, 51 (43–57) years; 57.9% male) who underwent 24-hour urine electrolyte measurement. Participants were grouped by the median UNa/UK ratio (4.40) for subsequent analysis, with 605 participants in each group. Additionally, we performed two-sample MR analyses to evaluate causal relationships between the UNa/UK ratio and HTN, circulating inflammatory proteins and immune cells, GM, and plasma metabolites.

**Results:**

A cross-sectional analysis revealed significant associations between the UNa/UK ratio and HTN prevalence, inflammation scores, and metabolites. Logistic regression confirmed the UNa/UK ratio as an independent predictor of HTN (odds ratio (OR): 1.076; 95% confidence interval (CI): 1.037–1.116). Spearman correlation analysis showed a positive correlation between the UNa/UK ratio and several inflammatory scores. The MR analyses indicated a causal effect of the UNa/UK ratio on HTN (inverse-variance weighted method: OR: 1.5130, 95% CI: 1.1613–1.9712), inflammatory proteins, immune cells, GM, and plasma metabolites.

**Conclusions:**

The UNa/UK ratio was significantly associated with HTN risk, systemic inflammation, GM dysbiosis, and metabolic disorders. Integrating both cross-sectional and MR approaches, our findings highlight the UNa/UK ratio as a clinically relevant biomarker and reinforce the role of dietary sodium–potassium balance in modulating HTN through underlying mechanisms involving inflammation, GM alterations, and metabolites.

## 1. Introduction

Hypertension (HTN) is a globally prevalent public health challenge [1], which results from a complex interplay of genetic predisposition, inflammation, metabolic dysregulation, gut microbiota (GM), and lifestyle factors [2, 3, 4, 5, 6]. Dietary sodium intake significantly influences the regulation of blood pressure, with excessive sodium consumption being a significant contributor to HTN and cardiovascular events [7, 8]. International health guidelines recommend reducing sodium intake to improve cardiovascular health and emphasize a balanced diet that is low in sodium and rich in potassium [9].

Chronic inflammation has been pathogenetically linked to the pathogenesis of HTN, fostering a sustained pro-inflammatory environment that impairs vascular function and elevates cardiovascular risk [10, 11]. Previous studies have shown that high salt intake promotes inflammation and disrupts immune homeostasis, thereby contributing to the pathogenesis of both HTN and cardiovascular disease [12], while a potassium-rich diet may offset the impact of high salt intake on HTN [13]. Emerging evidence also suggests that a high-salt diet can alter GM composition and its metabolic activity, potentially influencing HTN-related outcomes [14]. Wilck *et al*. [15] demonstrated that high salt intake reduces intestinal *Lactobacillus* abundance and increases pro-inflammatory Th17 cells. Therefore, the GM dysbiosis caused by imbalanced sodium and potassium intake is increasingly regarded as the core mechanism driving chronic inflammation and leading to the development of HTN.

Accurately quantifying sodium and potassium intake is fundamental to understanding their influence on HTN. Methods commonly used include dietary recall, food frequency questionnaires, spot urine sampling, and 24-hour urinary excretion. Among these, 24-hour urinary excretion is widely regarded as the most precise, as it closely reflects actual electrolyte intake [16, 17]. Notably, the 24-hour urinary sodium-to-potassium (UNa/UK) ratio has been recognized as a valuable indicator, providing a more comprehensive evaluation of sodium-potassium intake balance while minimizing variations due to urine volume and body weight [18].

Previous studies have demonstrated an association between a high-sodium, low-potassium diet and HTN, as well as inflammation, GM dysbiosis and metabolic disorders, however, the underlying causal mechanisms remain unclear. We hypothesize that the UNa/UK ratio is associated with HTN risk, and this relationship is mediated by systemic inflammation, GM alterations, and metabolic abnormalities. To address this hypothesis, we first conducted a cross-sectional analysis to examine whether there is an association between the UNa/UK ratio and HTN prevalence, as well as its related inflammatory scores and metabolites. Secondly, we explored the potential causality underlying these associations through two-sample Mendelian randomization (MR) analyses [19], based on genetic effect estimates derived from publicly accessible genome-wide association studies (GWAS). This method employs genetic variants as instrumental variables (IVs) to minimize confounding, reduce reverse causation bias, and strengthen the validity of causal interpretations.

## 2. Methods

### 2.1 Cross-Sectional Analysis

#### 2.1.1 Study Population

**Fig. 1.**
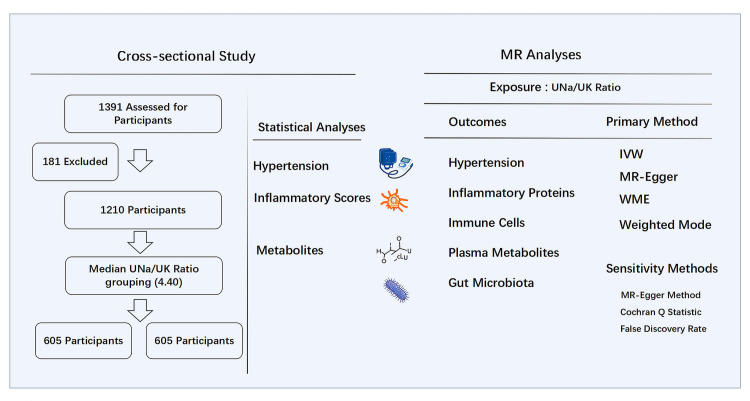
**Study design of cross-sectional study and MR analyses**. MR, Mendelian randomization; UNa/UK ratio, urinary sodium-to-potassium ratio; IVW, inverse-variance weighted; WME, weighted median estimator.

We retrospectively analyzed patients aged 18 to 65 who were hospitalized at the Second Hospital of Dalian Medical University from June 2014 to June 2024. A total of 1391 participants with complete clinical records and documented 24-hour urinary electrolyte measurements were included in the study. Patients were excluded based on the following conditions: (1) secondary HTN, such as pheochromocytoma, primary aldosteronism, or Cushing’s syndrome; (2) acute myocardial infarction or heart failure with symptoms falling under New York Heart Association classification stage III or IV; (3) chronic kidney disease stage 3–5; (4) current use of medications known to significantly alter electrolyte homeostasis. A total of 1210 patients were retained for the final analysis. The study design is illustrated in Fig. 1. Ethical approval for the study was granted by the Ethics Committee of the Second Hospital of Dalian Medical University, with the approval number: KY2025-188-01-01, and all procedures complied with the Declaration of Helsinki.

#### 2.1.2 Assessment

We collected 24-hour urine samples from patients to assess sodium and potassium excretion. After hospital admission, participants were guided to begin 24-hour urine collection starting from the first morning void on the second day until the first morning void of the following day using a designated container. Trained staff provided detailed instructions on the collection procedure. After the full 24-hour sampling had concluded, the total 24-hour urine volume was measured and recorded.

We calculated the UNa/UK ratio using 24-hour urinary sodium and potassium concentrations. Since 24-hour urinary sodium excretion is considered the most accurate method for evaluating dietary salt intake [20], we estimated the 24-hour salt intake using the following formula: Salt intake (g/24 h) = urinary sodium concentration (mmol/L) × 24-hour urine volume (L) × 0.0585 [21].

Basic patient information, including age, sex, history of HTN and diabetes, and laboratory test results was obtained from hospital records. Upon admission, blood pressure was measured using an electronic sphygmomanometer after 20 minutes of rest. HTN was defined as having a recorded medical history of the condition, current use of antihypertensive therapy, or elevated blood pressure at admission (systolic >140 mmHg or diastolic >90 mmHg). Laboratory tests included blood cell counts, lipid-related measures, liver and kidney function panels, fasting serum glucose, circulating amino acids, and other relevant biomarkers. Hepatic steatosis was diagnosed via ultrasonography.

The inflammatory scores were calculated using the following formulas: neutrophil-to-lymphocyte ratio (NLR) [22] = neutrophil count (×10^9^/L)/lymphocyte count (×10^9^/L); systemic immune-inflammation index (SII) [23] = platelet count (×10^9^/L) × neutrophil count (×10^9^/L)/lymphocyte count (×10^9^/L); systemic inflammation response index (SIRI) [24] = neutrophil count (×10^9^/L) × monocyte count (×10^9^/L)/lymphocyte count (×10^9^/L).

#### 2.1.3 Statistical Analyses

The participants were stratified into two groups according to the median UNa/UK ratio for subsequent analysis. Normality of the data was initially evaluated using the Shapiro–Wilk test. Normally distributed continuous variables were expressed as mean ± standard deviation. Differences between groups were assessed using an independent samples *t*-test. Non-normally distributed continuous variables were reported as median and interquartile range, with group differences assessed using the Mann-Whitney U test. Categorical variables were presented as frequencies (n) and percentages (%), and differences were assessed using the chi-square test. To examine the association between HTN and the UNa/UK ratio, we conducted binary logistic regression analysis. Adjustments were made for age, sex, and other relevant covariates in the models to account for potential confounders. Finally, we performed Spearman correlation analysis to evaluate the relationship between inflammatory scores and the UNa/UK ratio as well as other metabolic indicators. Statistical significance was defined as *p* < 0.05. We conducted all cross-sectional analyses using SPSS software, version 27.0 (IBM Corp., Armonk, NY, USA).

### 2.2 Mendelian Randomization Analyses

#### 2.2.1 Study Design of MR

The study design is illustrated in Fig. 1. To investigate possible causal associations, we applied two-sample MR analyses examining the relationship between the UNa/UK ratio and HTN, inflammatory proteins, immune cells, plasma metabolites, and GM. We also applied a reverse MR analysis, treating HTN as the exposure and the UNa/UK ratio as the outcome, to determine whether HTN exerts a causal effect on the UNa/UK ratio. The genome-wide association studies (GWAS) data used in this MR analyses are publicly available, as shown in **Supplementary Table 1**. All MR analyses followed the Strengthening the Reporting of Observational Studies in Epidemiology using Mendelian Randomization reporting guidelines, and we adopted several methods to follow the three fundamental assumptions of MR [25].

#### 2.2.2 Date Sources

2.2.2.1 UNa/UK Ratio and HTN

We utilized the GWAS summary statistics for the UNa/UK ratio from the study conducted by Zanetti *et al*. [26], which analyzed data from 326,938 participants in the UK Biobank. We accessed GWAS summary data for essential HTN from FinnGen R11 release, which includes 116,714 cases and 316,345 controls. The FinnGen dataset was analyzed using Scalable and Accurate Implementation of GEneralized mixed model (SAIGE), a generalized mixed model association test that applies saddlepoint approximation to correct for case-control imbalance, with adjustments for sex, age, the first ten principal components, and genotyping batch. Classification of cases and controls was based on hospital records coded according to the 10th revision of the International Classification of Diseases (ICD-10), with the most recent data update in June 2024 (https://www.finngen.fi/en).

2.2.2.2 Circulating Inflammatory Proteins, Immune Cells, Plasma Metabolites, and GM

We obtained the GWAS summary statistics for the protein quantitative trait loci of 91 circulating inflammatory proteins, as reported by Zhao *et al*. [27], which included 14,824 participants. We also acquired summary statistics for 731 immune cell traits from a large-scale immune cell study conducted by Orrù *et al*. [28], based on a cohort of 3757 Sardinians. Additionally, we retrieved genetic data from a GWAS involving 1091 individual blood metabolites and 309 calculated metabolite ratios [29].

To ensure the robustness and comprehensiveness of our study, we selected four GWAS datasets on GM as genetic instruments. As the first dataset, we leveraged summary statistics from the MiBioGen consortium (https://mibiogen.gcc.rug.nl), which is currently the most comprehensive database of genetic influences on human GM [30]. The study included 18,340 individuals from 24 cohorts, of which 78% were Europeans. A total of 211 taxa were included. Additionally, we incorporated GWAS summary statistics from three independent GM studies conducted in European populations [31, 32, 33].

#### 2.2.3 Data Extraction

To ensure the validity of our conclusions, the IVs in the MR analyses followed specific criteria. First, single nucleotide polymorphisms (SNPs) were selected based on the standard genome-wide significance cutoff of *p* < 5×10^-8^. For GM taxa, we adopted a more relaxed threshold (*p* < 5×10^-5^) to increase sensitivity and capture more potential associations, consistent with previous MR studies. Second, we performed linkage disequilibrium (LD) clumping for all selected genetic variants at an r^2^ <0.001 threshold within ± 10,000 kilobases using the 1000 genomes reference panel. Additionally, to minimize the risk of weak instrument bias, we assessed the F-statistics of selected IVs, with an F-value <10 indicating a weak IV, which was subsequently excluded [34].

#### 2.2.4 Statistical Analyses

We employed two-sample MR analyses to explore whether the UNa/UK ratio exerts a causal influence on HTN and a spectrum of biological components such as inflammatory proteins, immune cells, metabolic profiles, and GM taxa. The primary MR approach employed was the inverse-variance weighted (IVW) method [35]. The fixed-effects model was used when there was no substantial heterogeneity among genetic instruments, while the multiplicative random-effects model accounted for potential heterogeneity, providing more robust estimates across different scenarios. To enhance robustness, we also performed MR-Egger regression [36], weighted median estimator (WME) [37], and weighted mode methods [38]. The IVW method was considered the most powerful.

To enhance the robustness of our findings, several sensitivity analyses were conducted to assess the reliability of effect estimates. We assessed horizontal pleiotropy using the MR-Egger method [36]. *p* < 0.05 in the Egger intercept test indicates the presence of horizontal pleiotropy, and such results were excluded. We also used Cochran Q statistic to assess heterogeneity [39]. Q statistics significant at *p* < 0.05 can imply the presence of heterogeneity. Our results were corrected for multiple hypothesis testing using the Hochberg false discovery rate (FDR). Associations were considered statistically significant if both P-IVW <0.05 and P-FDR <0.1. If P-IVW <0.05 but P-FDR >0.1, the results were classified as suggestive associations based on previous analyses [40]. MR analyses were implemented in R software (v4.3.2; https://www.r-project.org/), utilizing the “TwoSampleMR” (https://mrcieu.github.io/TwoSampleMR/) and “MR-PRESSO” (https://github.com/rondolab/MR-PRESSO) libraries.

## 3. Results

### 3.1 Baseline Characteristics

Table 1 presents the baseline characteristics of the 1210 participants. The median age was 51 (43–57) years, and 700 individuals (57.9%) were male. Participants were categorized into two groups based on the median UNa/UK ratio of 4.40. There were significant differences between the two groups in terms of sex, diastolic blood pressure (DBP), HTN prevalence, salt intake, aspartate aminotransferase/alanine aminotransferase (AST/ALT) ratio, lipid profile, and amino acid levels. Participants with a higher UNa/UK ratio exhibited significantly elevated inflammatory scores, including NLR, SII, and SIRI. The remaining metabolic indicators are presented in **Supplementary Table 2**.

**Table 1.** **Baseline characteristics of the participants**.

Variables	UNa/UK ratio <4.40 (n = 605)	UNa/UK ratio >4.40 (n = 605)	*p*-value
Age, years	52 (44, 57)	51 (42, 57)	0.129
Male, n (%)	326 (53.9%)	374 (61.8%)	0.005
HTN, n (%)	436 (72.1%)	490 (81.0%)	<0.01
Diabetes, n (%)	226 (37.4%)	239 (39.5%)	0.442
Fatty liver, n (%)	361 (59.7%)	377 (62.3%)	0.346
SBP, mmHg	140 (127, 157)	143 (130, 156)	0.110
DBP, mmHg	89 (81, 100)	92 (84, 102)	0.005
Urine volume/24 h, L	1.94 (1.40, 2.50)	2.00 (1.50, 2.65)	0.002
UNa/24 h, mmol/L	88.82 (61.85, 121.79)	167.55 (116.87, 226.95)	<0.01
UK/24 h, mmol/L	36.77 (26.49, 49.25)	20.89 (14.92, 28.48)	<0.01
Salt intake, g	9.13 (6.32, 13.51)	17.83 (11.96, 30.43)	<0.01
NLR	1.75 (1.36, 2.36)	1.87 (1.43, 2.40)	0.013
SII	407.53 (291.29, 566.86)	437.66 (324.00, 593.21)	0.015
SIRI	0.66 (0.46, 0.97)	0.72 (0.50, 1.05)	0.023
Fasting glucose, mmol/L	5.58 (5.07, 6.81)	5.66 (5.10, 7.20)	0.280
AST, U/L	20.70 (17.09, 26.80)	20.54 (16.79, 26.15)	0.436
ALT, U/L	24.26 (16.89, 35.40)	24.13 (17.86, 36.17)	0.495
AST/ALT	0.88 (0.69, 1.08)	0.83 (0.67, 1.03)	0.028
Bile acid, µmol/L	3.30 (1.97, 5.42)	3.39 (2.14, 5.60)	0.378
Creatinine, µmol/L	63.87 (53.68, 77.10)	64.61 (52.87, 77.00)	0.994
Uric acid, µmol/L	368.70 (296.64, 443.76)	367.42 (302.76, 443.78)	0.873
TC, mmol/L	5.06 (4.41, 5.73)	4.92 (4.23, 5.65)	0.023
TG, mmol/L	1.63 (1.13, 2.37)	1.66 (1.20, 2.52)	0.190
LDL-C, mmol/L	2.90 (2.38, 3.47)	2.83 (2.20, 3.45)	0.097
HDL-C, mmol/L	1.12 (0.95, 1.34)	1.08 (0.94, 1.28)	0.015
Glycine, µmol/L	158.35 (140.16, 180.57)	164.99 (149.01, 186.16)	<0.01
Glutamate, µmol/L	117.50 (102.98, 138.75)	123.42 (107.20, 139.94)	0.009
Proline, µmol/L	364.88 (290.27, 455.15)	375.38 (302.85, 478.99)	0.033
Glutamate/Glutamine	10.82 (8.03, 14.86)	11.21 (8.63, 16.29)	0.019

Abbreviations: UNa/UK ratio, urinary sodium-to-potassium ratio; UK/24 h, 24-hour urinary potassium excretion; UNa/24 h, 24-hour urinary sodium excretion; SBP, systolic blood pressure; DBP, diastolic blood pressure; NLR, neutrophil-to-lymphocyte ratio; SII, systemic inflammation index; SIRI, systemic inflammation response index; AST, aspartate aminotransferase; ALT, alanine aminotransferase; TC, total cholesterol; TG, triglycerides; LDL-C, low-density lipoprotein cholesterol; HDL-C, high-density lipoprotein cholesterol.

### 3.2 Associations of the UNa/UK Ratio and HTN

To assess the association between the UNa/UK ratio and HTN, we conducted binary logistic regression analysis (Table 2). In the crude model, the UNa/UK ratio (odds ratio [OR] 1.076, 95% confidence interval [CI] 1.037–1.116) and 24-hour urinary sodium excretion (UNa/24 h) (OR 1.006, 95% CI 1.004–1.008) were identified as independent predictors of HTN. Notably, the UNa/UK ratio exhibited a stronger magnitude of association with HTN than UNa/24 h, as reflected by its higher OR per unit change. These two indicators remained significant predictors of HTN after adjusting for age, sex, serum potassium, serum sodium, diabetes, and creatinine. In contrast, 24-hour urinary potassium excretion (UK/24 h) was not significantly associated with HTN.

**Table 2.** **Associations of the UNa/UK ratio and HTN**.

Variable		OR (95% CI)	*p*-value
UNa/UK ratio			
	Crude	1.076 (1.037, 1.116)	<0.01
	Mode1	1.075 (1.036, 1.117)	<0.01
	Mode2	1.070 (1.030, 1.111)	<0.01
UNa/24 h			
	Crude	1.006 (1.004, 1.008)	<0.01
	Mode1	1.006 (1.003, 1.008)	<0.01
	Mode2	1.005 (1.003, 1.007)	<0.01
UK/24 h			
	Crude	1 (0.992, 1.007)	0.906

Notes: Crude: unadjusted model; Model 1: adjusted for age and gender; Model 2: adjusted for age, gender, serum potassium, serum sodium, diabetes, and creatinine. Collinearity among variables in the Model 2 was evaluated using variance inflation factor, with all variance inflation factor values <5 indicating no significant collinearity (**Supplementary Table 3**). Abbreviations: UNa/UK ratio, urinary sodium-to-potassium ratio; HTN, hypertension; UK/24 h, 24-hour urinary potassium excretion; UNa/24 h, 24-hour urinary sodium excretion; OR, odds ratio; CI, confidence interval.

### 3.3 Correlation of Inflammatory Score With Clinical Parameters

In the bivariate correlation analysis conducted across the whole cohort via Spearman correlation analysis, the inflammatory scores NLR, SII, and SIRI showed a positive correlation with the UNa/UK ratio, blood pressure, heart rate (HR) and glutamate levels, while NLR and SIRI exhibited a significant positive correlation with salt intake (Table 3).

**Table 3.** **Correlation of inflammatory score with clinical parameters**.

Variables	NLR		SII		SIRI	
	r	*p*-value	r	*p*-value	r	*p*-value
UNa/UK ratio	0.075	0.009	0.073	0.011	0.071	0.013
Salt intake	0.081	0.005	0.051	0.075	0.066	0.021
Age	–0.055	0.057	–0.141	<0.01	–0.132	<0.01
HR	0.086	0.003	0.160	<0.01	0.130	<0.01
SBP	0.126	<0.01	0.124	<0.01	0.116	<0.01
DBP	0.122	<0.01	0.142	<0.01	0.130	<0.01
AST/ALT	–0.037	0.200	–0.055	0.056	–0.117	<0.01
TC	–0.104	<0.01	–0.017	0.557	–0.074	0.010
TG	0.009	0.766	0.032	0.272	0.068	0.017
LDL-C	–0.0078	0.006	<0.01	0.990	–0.040	0.159
HDL-C	–0.029	0.314	–0.011	0.689	–0.117	<0.01
Glycine	0.004	0.880	–0.001	0.977	–0.046	0.113
Glutamate	0.086	0.003	0.132	<0.01	0.094	0.001
Proline	0.052	0.070	0.046	0.111	0.040	0.164

Abbreviations: UNa/UK ratio, urinary sodium-to-potassium ratio; NLR, neutrophil-to-lymphocyte ratio; SII, systemic inflammation index; SIRI, systemic inflammation response index; HR, heart rate; SBP, systolic blood pressure; DBP, diastolic blood pressure; AST/ALT, aspartate aminotransferase to alanine aminotransferase ratio; TC, total cholesterol; TG, triglycerides; LDL-C, low-density lipoprotein cholesterol; HDL-C, high-density lipoprotein cholesterol.

### 3.4 Bidirectional MR Analyses of the UNa/UK Ratio and HTN

After removing confounding factors and outliers, a total of 15 SNPs were included in the final analysis. We found a causal effect of the UNa/UK ratio on risk of HTN in our MR analysis (IVW: OR 1.5130, 95% CI: 1.1613–1.9712, *p* = 0.0022), as shown in Table 4. No evidence of pleiotropy was detected based on the MR-Egger intercept test (*p* = 0.649). Other sensitivity analyses are provided in **Supplementary Table 4**. The reverse MR analysis did not reveal a causal relationship from HTN to the UNa/UK ratio (**Supplementary Table 5**).

**Table 4.** **MR analysis of the UNa/UK ratio on HTN**.

Exposure	Outcome	Method	nSNP	Beta	SE	OR	OR (95% CI)	*p*-value
UNa/UK ratio	HTN	IVW	15	0.4141	0.1350	1.5130	1.1613–1.9712	0.0022
		MR-Egger	15	0.7388	0.7097	2.0934	0.5209–8.4139	0.3169
		Weighted median	15	0.3538	0.1552	1.4245	1.0509–1.9309	0.0226
		Weighted mode	15	0.2307	0.3324	1.2595	0.6565–2.4164	0.4991

Abbreviations: MR, Mendelian randomization; UNa/UK ratio, urinary sodium-to-potassium ratio; HTN, hypertension; IVW, inverse-variance-weighted; nSNP, number of single nucleotide polymorphisms; SE, standard error; OR, odds ratio; CI, confidence interval.

### 3.5 Causal Effects of the UNa/UK Ratio on Inflammatory Proteins and Immune Cells

**Fig. 2.**
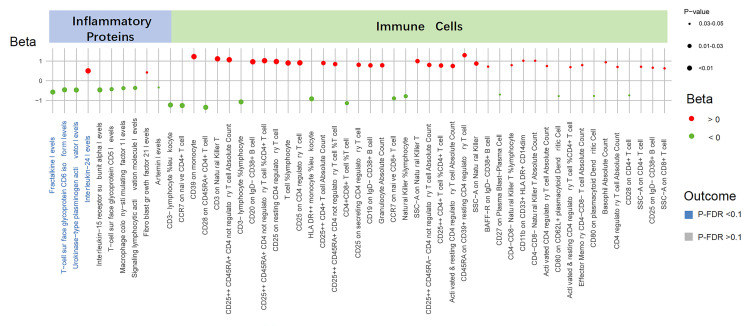
**Causal effects of the UNa/UK ratio on inflammatory proteins and immune cells**. This figure displays the associations between the UNa/UK ratio and inflammatory proteins and immune cells. Each dot represents a trait, with color indicating the direction of effect (red for Beta >0, green for Beta <0), and size reflecting the *p*-value. Associations were considered significant when P-IVW <0.05 and P-FDR <0.1 (blue labels), and suggestive when P-FDR >0.1 (gray labels). Beta values were estimated using IVW method. UNa/UK ratio, urinary sodium-to-potassium ratio; IVW, inverse-variance weighted; FDR, false discovery rate.

The summary of causal relationships between the UNa/UK ratio and circulating inflammatory proteins and immune cell populations is presented in Fig. 2. We identified ten inflammatory proteins influenced by the UNa/UK ratio (**Supplementary Table 6**). Of these proteins, four demonstrated statistically significant associations (P-IVW <0.05, P-FDR <0.1), whereas the remaining six proteins exhibited suggestive associations (P-IVW <0.05, P-FDR >0.1). Specifically, a higher UNa/UK ratio was significantly associated with decreased levels of Fractalkine (IVW: OR 0.564, 95% CI 0.386–0.826, *p* = 0.003), cluster of differentiation (CD) 6 (IVW: OR 0.632, 95% CI 0.465–0.860, *p* = 0.003), and urokinase-type plasminogen activator (IVW: OR 0.626, 95% CI 0.458–0.857, *p* = 0.003). Conversely, a positive correlation was observed between the UNa/UK ratio and elevated interleukin (IL)-24 levels (IVW: OR 1.656, 95% CI 1.165–2.354, *p* = 0.005). Detailed sensitivity analyses are provided in **Supplementary Table 7**.

Furthermore, utilizing the IVW method, we identified 43 immune cell counts and cell ratios that are influenced by the UNa/UK ratio (Fig. 2 and **Supplementary Table 8**). Significant associations involved multiple immune cell subsets, including T cells, B cells, monocytes, natural killer cells, and dendritic cells. Nevertheless, after FDR correction, all *p*-values exceeded 0.1, suggesting that these are suggestive associations (**Supplementary Table 9**).

### 3.6 Causal Effects of the UNa/UK Ratio on Plasma Metabolites

**Fig. 3.**
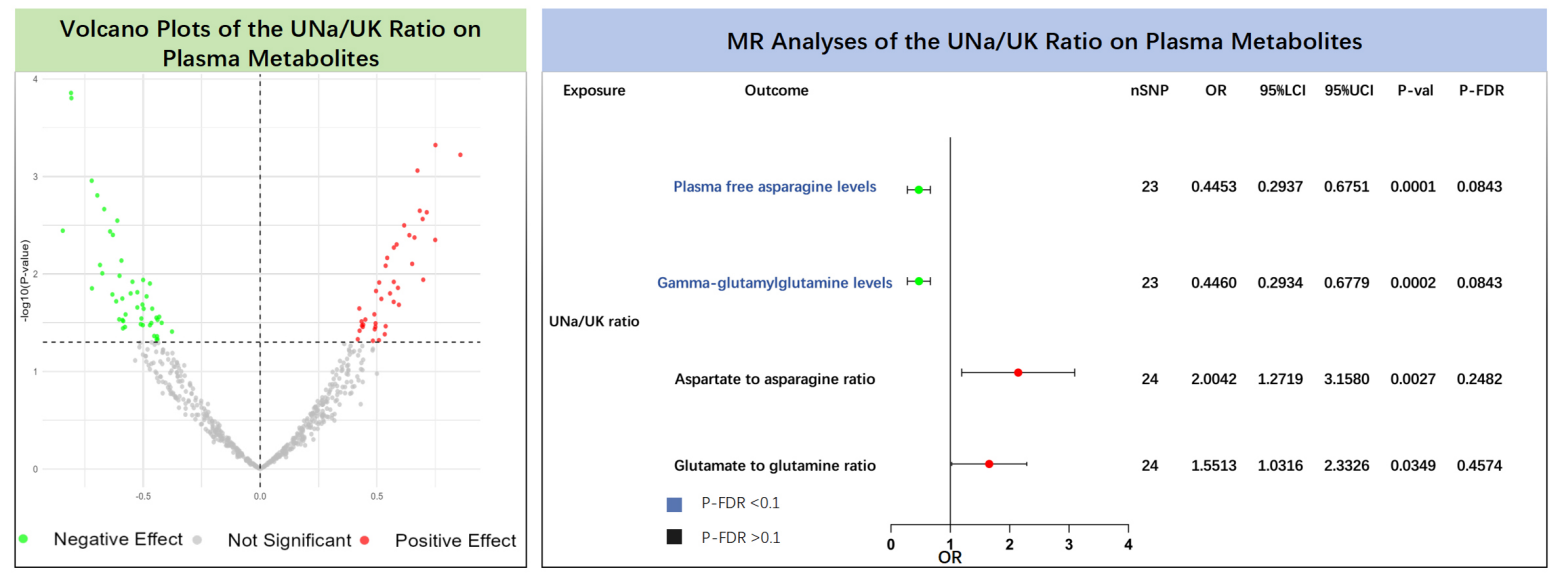
**Causal effects of the UNa/UK ratio on plasma metabolites**. The left panel shows the volcano plot illustrating the effects of the UNa/UK ratio on plasma metabolites using the inverse variance weighted method. The right panel shows the forest plot of amino acids and their ratios that showed significant positive associations. MR, Mendelian randomization; UNa/UK ratio, urinary sodium-to-potassium ratio; FDR, false discovery rate; OR, odds ratio; nSNP, number of single nucleotide polymorphisms.

The results of the MR analyses showed that plasma free asparagine levels (IVW: OR = 0.445, 95% CI: 0.294–0.675, *p* = 0.0001, P-FDR = 0.084) and gamma-glutamylglutamine levels (IVW: OR = 0.446, 95% CI: 0.293–0.678, *p* = 0.0002, P-FDR = 0.084) were negatively associated with the UNa/UK ratio (**Supplementary Table 10**, Fig. 3). Even after correcting for multiple testing via the FDR approach, the associations retained statistical significance (P-FDR <0.1), suggesting robust evidence for their potential causal relationship. Additionally, we identified 80 independent metabolic traits that exhibited suggestive associations (P-IVW <0.05, P-FDR >0.1) with the UNa/UK ratio (**Supplementary Table 11**, Fig. 3). Among these, the UNa/UK ratio was positively associated with the glutamate/glutamine ratio (IVW: OR = 2.004, 95% CI: 1.272–3.158, *p* = 0.0027, P-FDR = 0.2482), consistent with findings from cross-sectional analyses showing elevated glutamate/glutamine ratios among participants with higher UNa/UK ratios. Detailed sensitivity analyses are provided in **Supplementary Table 12**.

### 3.7 Causal Effects of the UNa/UK Ratio on GM

**Fig. 4.**
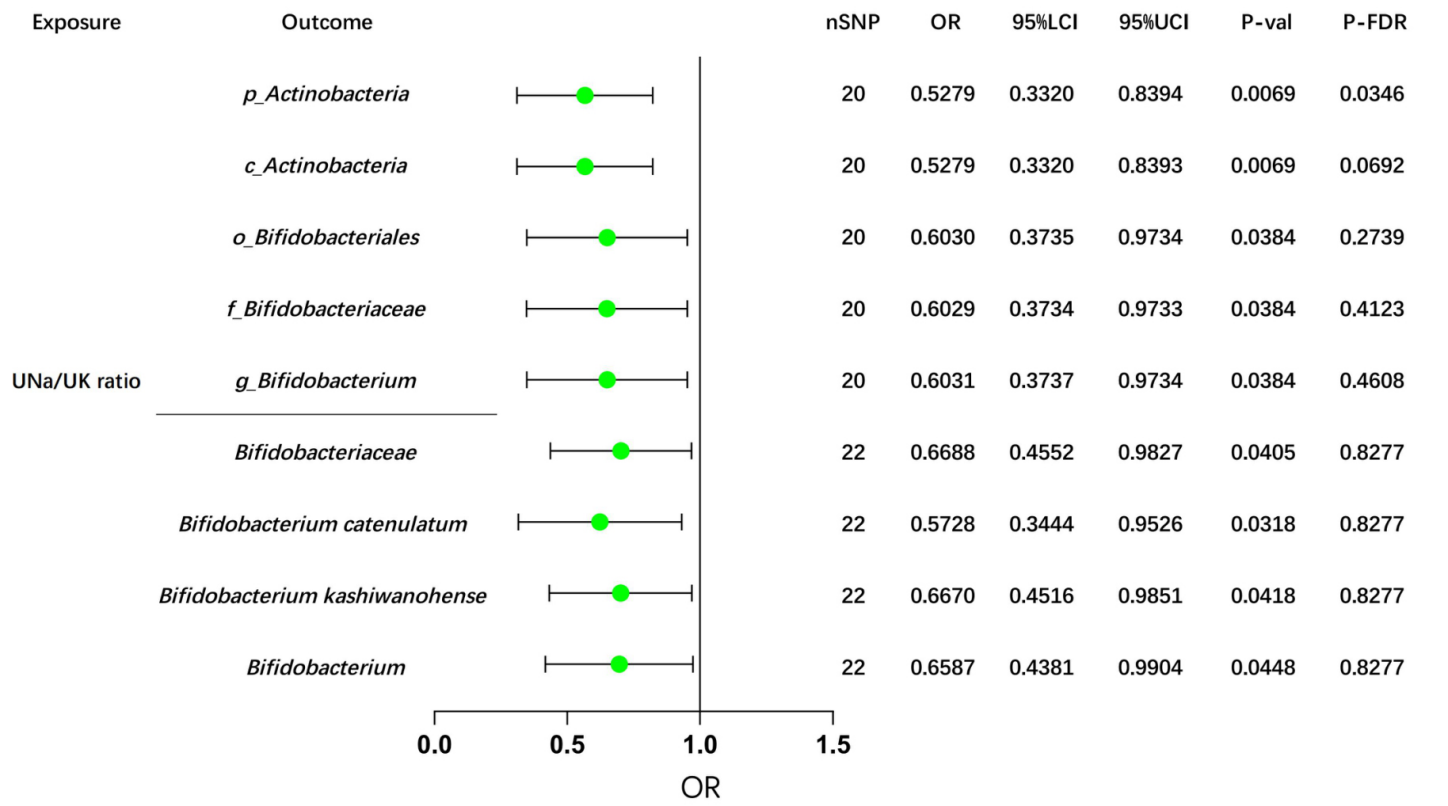
**MR analyses of the UNa/UK ratio on *Bifidobacterium* using the IVW method**. This forest plot illustrates the causal effects between the UNa/UK ratio and *Bifidobacterium* and its related upstream and downstream taxa, using the IVW method. MR, Mendelian randomization; UNa/UK ratio, urinary sodium-to-potassium ratio; IVW, inverse-variance weighted; OR, odds ratio; LCI, lower confidence interval; UCI, upper confidence interval; nSNP, number of single nucleotide polymorphisms; FDR, false discovery rate. The prefix “p_/c_/o_/f_/g_” represents phylum/class/order/family/genus respectively.

In the Dutch GM study, after FDR correction, our MR analyses revealed potential causal effects of the UNa/UK ratio on the abundance of two GM taxa: *p_Actinobacteria* (IVW: OR = OR= 0.5279, 95% CI: 0.3320–0.8394, *p* = 0.0069) and *c_Actinobacteria* (IVW: OR = 0.5279, 95% CI: 0.3320–0.8393, *p* = 0.0069), as shown in **Supplementary Table 13** and Fig. 4.

Consistently, we also observed negative associations between the UNa/UK ratio and *Bifidobacterium*, taxa downstream of *Actinobacteria*, as well as their related taxa, in the GWAS data from the Dutch Microbiome Project and the FINRISK study (Fig. 4). Additionally, several suggestive associations were identified and summarized in **Supplementary Table 14**. Detailed sensitivity analyses are provided in **Supplementary Table 15**.

## 4. Discussion

In this cross-sectional study, we found that the UNa/UK ratio was associated with HTN, exhibiting a stronger correlation than urinary sodium or potassium alone, and was also related to inflammatory scores and metabolic indicators. MR analyses further supported a potential causal effect of the UNa/UK ratio on HTN. Additionally, the UNa/UK ratio was significantly associated with four inflammatory proteins, two plasma metabolites, and two GM taxa, alongside several suggestive associations.

The benefits of a low-salt diet in reducing HTN risk have been widely recognized, and latest guidelines further emphasize the importance of increasing potassium intake alongside sodium reduction [41]. Consistently, after adjustment for multiple confounding variables, a significant link remained between elevated UNa/UK levels and hypertension risk (OR = 1.070, 95% CI: 1.030–1.111, *p* < 0.01). In line with previous studies [26, 42], our study provides further evidence supporting the importance of the UNa/UK ratio, which reflects dietary sodium and potassium intake, in HTN risk assessment. These findings suggest that the UNa/UK ratio may be a more reliable indicator of HTN risk than sodium levels alone, highlighting the importance of maintaining a balanced intake of sodium and potassium for effective blood pressure management.

Previous studies have shown that a high-sodium diet can promote inflammation by activating immune cells, increasing oxidative stress, impairing endothelial function, and other related mechanisms [43, 44, 45]. Ferguson *et al*. [14] discovered that a high-sodium diet promotes inflammation and HTN by altering the GM and activating dendritic cells, which subsequently increase the formation of immunogenic isolevuglandin adducts and cytokine production. Kleinewietfeld *et al*. [46] demonstrated that a high-sodium diet enhances the differentiation of CD4^+^ T cells into Th17 cells, which produce IL-17, a cytokine that drives inflammation and the pathogenesis of HTN. Moreover, a cross-sectional study also found that potassium intake may have protective anti-inflammatory effects, as it was shown to negatively correlate with pro-inflammatory mediators. Our analyses also revealed a positive association between the UNa/UK ratio and inflammatory scores, while MR analyses identified a significant association between the UNa/UK ratio and various inflammatory proteins and immune cells, suggesting that a high-sodium, low-potassium diet may influence inflammatory responses through these factors, highlighting the potential role of dietary sodium and potassium balance in modulating immune responses and inflammation.

Dietary habits not only influence the onset and progression of HTN but also exert a complex and intricate impact by altering the composition of GM, the production of metabolites, and their interactions. Numerous animal and human studies have further confirmed and reinforced the evidence that a high-sodium diet induces significant alterations in GM [15, 47, 48]. Additionally, research demonstrated that fecal microbiota transplantation from HTN patients to germ-free mice led to elevated blood pressure [49]. The Mediterranean dietary pattern emphasizes abundant consumption of plant-based foods and unsaturated fats, including fruits, vegetables, whole grains, and olive oil, along with relatively low sodium and high potassium levels, has been shown to induce changes in *Bifidobacterium* and *Lactobacillus* within the GM [50]. Our study found that the UNa/UK ratio was significantly negatively associated with *Actinobacteria* phylum and *Actinobacteria* class from the Dutch Microbiome Project. Downstream taxa, including the *Bifidobacteriales* order, *Bifidobacteriaceae* family, and *Bifidobacterium* genus, showed significant associations with the UNa/UK ratio, although the FDR-adjusted *p*-values were greater than 0.1. In the analyses of the GM database from Finland, a negative correlation was also found between the UNa/UK ratio and *Bifidobacterium*. These findings suggest that a high-sodium, low-potassium diet may result in the depletion of *Bifidobacterium*, potentially contributing to dysbiosis and impairing gut health, which could further exacerbate HTN and related metabolic disorders.

Another key finding of this study is the significant association between the UNa/UK ratio and metabolic abnormalities, including alterations in lipid profiles, amino acid metabolism, and other essential metabolites. Specifically, the UNa/UK ratio was significantly associated with glutamate levels and the glutamate/glutamine ratio, and this association was further supported by subsequent MR analyses. Glutamate is a non-essential amino acid that is essential for neurotransmission, metabolism, and immune function, but excessive levels can cause excitotoxicity, oxidative stress, and metabolic imbalance [51, 52, 53]. Zheng *et al*. [54] found that in rats, a high-sodium diet reshapes the GM and disrupts glutamate–glutamine metabolism by elevating glutamic acid and its derivatives. In several clinical studies, elevated circulating glutamate levels have been associated with an increased risk of cardiovascular and cerebrovascular diseases, including stroke, coronary artery disease, and subarachnoid hemorrhage [55, 56, 57]. The elevated glutamate levels and increased glutamate/glutamine ratio observed in the high UNa/UK group in this study suggest a potential mechanistic link between dietary sodium-potassium imbalance, glutamate metabolism disturbances, and the pathogenesis of HTN. This study also identified abnormalities in other amino acids and their metabolites, which are hypothesized to be linked to inflammation, GM alterations, and other potential mechanisms. Further research is needed to elucidate the underlying pathways and confirm these associations.

This study represents a novel attempt to explore the association between the UNa/UK ratio and HTN, inflammatory markers, GM, and metabolites. Through cross-sectional analysis, we examined the association between the UNa/UK ratio and HTN, along with related factors. Furthermore, we conducted MR analyses to validate and extend these causal relationships, effectively minimizing potential confounding biases. Our findings provide robust evidence for the causal relationship between the UNa/UK ratio, a key indicator reflecting sodium and potassium intake balance, and various metabolic factors.

However, our study has some limitations. The cross-sectional study was conducted in an Asian population, while the MR analyses were based on a European cohort. Further studies involving more diverse populations are warranted to enhance the external validity and generalizability of these findings. Additionally, while the cross-sectional study provided preliminary association analysis, the modest sample size may restrict the statistical power and limit the extent to which the findings can be generalized. Therefore, future studies should involve large-scale prospective cohort studies to confirm these associations. Moreover, due to sample size constraints, we were unable to conduct age-stratified subgroup analyses to explore potential heterogeneity in the associations between the UNa/K ratio and the outcomes of interest across different age groups. Furthermore, as the present study used a retrospective design without collecting fecal samples for GM sequencing, direct analysis of the clinical association between the UNa/K ratio and GM in the included clinical population was not possible.

## 5. Conclusions

This study demonstrates a strong association between the UNa/UK ratio and HTN, inflammation, GM, and metabolic abnormalities. Compared to sodium levels alone, the UNa/UK ratio may serve as a more reliable indicator of HTN risk. A high-sodium, low-potassium diet may contribute to systemic inflammation, depletion of *Bifidobacterium*, and dysregulation of glutamate metabolism. Overall, this study reveals the complex interplay between dietary sodium-potassium balance, HTN, inflammation, GM and related metabolism, providing valuable insights for future mechanistic research and potential intervention strategies.

## Data Availability

All data generated or analysed during this study are included in this published article and its supplementary information files. Detailed information on the GWAS datasets used, including data sources and GWAS IDs, is provided in **Supplementary Table 1**.

## Abbreviations

BP, blood pressure; CI, confidence interval; CD, cluster of differentiation; DBP, diastolic blood pressure; FDR, false discovery rate; GM, gut microbiota; GWAS, genome-wide association; HR, heart rate; HTN, hypertension; ICD-10, international classification of diseases, 10th revision; IL, interleukin; IVs, instrumental variables; IVW, inverse-variance weighted; LD, linkage disequilibrium; MR, Mendelian randomization; NLR, neutrophil-to-lymphocyte ratio; OR, odds ratio; SII, systemic immune-inflammation index; SIRI, systemic inflammation response index; SNPs, single nucleotide polymorphisms; UNa/UK ratio, urinary sodium-to-potassium ratio; UK/24 h, 24-hour urinary potassium excretion; UNa/24 h, 24-hour urinary sodium excretion; WME, weighted median estimator.

## Supplementary Material

Supplementary material associated with this article can be found, in the online version, at https://doi.org/10.31083/RCM44058.
